# Survivorship Challenges in Metastatic Prostate Cancer in the Era of Prolonged Survival: A Review

**DOI:** 10.3390/cancers18152465

**Published:** 2026-07-31

**Authors:** Cristina Cano Garcia, Joseph Moryousef, Jehonathan H. Pinthus, Darryl P. Leong

**Affiliations:** 1 Population Health Research Institute, McMaster University and Hamilton Health Sciences, Hamilton, ON L8L 2X2, Canada; 2 Department of Surgery, McMaster University, Hamilton, ON L8S 4K1, Canada; 3 Department of Urology, University Hospital Frankfurt, Goethe University Frankfurt am Main, 60596 Frankfurt, Germany; 4 Department of Medicine, McMaster University and Hamilton Health Sciences, Hamilton, ON L8N 3Z5, Canada

**Keywords:** prostate cancer, survivorship, quality of life

## Abstract

In metastatic prostate cancer, there have been pronounced survival gains driven by the introduction of combination and intensification strategies. Consequently, many patients now experience prolonged survival with metastatic disease. While these advances represent a major therapeutic success, prolonged exposure to systemic therapies is associated with a broad spectrum of adverse effects. These side effects affect multiple domains, including bone health, cardiovascular risk, physical function, and psychological well-being. As a result, survivorship has emerged as a critical and increasingly relevant aspect of care in metastatic prostate cancer. In parallel, the importance of patient-reported outcomes in clinical trials, alongside traditional oncologic endpoints, is increasingly acknowledged. Overall, survivorship care in metastatic disease remains incompletely conceptualized as the long-term impact of cumulative treatment burden is often underrecognized. This narrative review examines key survivorship challenges in metastatic prostate cancer in an era of improving survival, with a focus on treatment-related toxicities, quality of life, and unmet needs in supportive care. We highlight current evidence, identify gaps in knowledge, and discuss future directions for integrating survivorship into routine clinical practice.

## Simple Summary

Recent advances in the treatment of metastatic prostate cancer have helped many patients live longer than ever before. As patients survive longer with their prostate cancer on long-term cancer treatment, it is becoming increasingly important to understand and manage their long-term sequelae. This review discusses some of the most common challenges faced by metastatic prostate cancer patients, including heart and bone health problems, loss of muscle strength, weight and body composition changes, hot flashes, emotional well-being, and overall quality of life. We also review the evidence behind supportive care measures. By addressing these issues through a team-based approach, the goal of healthcare providers is to preserve functional independence and quality of life while living with metastatic prostate cancer.

## 1. Introduction

Prostate cancer is the second most common cancer and the fifth leading cause of cancer death in men worldwide. In 2022, there were approximately 1,460,000 cases and 396,000 related deaths [1]. Based on data from a large population-based U.S. cancer registry, approximately 70% of prostate cancer cases are diagnosed with localized disease, 14% with regional lymph node involvement, and 8% with distant metastases (staging information was unavailable for approximately 8% of patients) [2]. However, metastatic prostate cancer remains a major contributor to cancer-related morbidity and mortality. While historically associated with limited survival, the therapeutic landscape has evolved substantially over the past decade [3]. The introduction of combination and intensification strategies with novel therapies has led to significant improvements in survival outcomes in both metastatic hormone-sensitive and castration-resistant disease. As a result, many patients now live for years with metastatic prostate cancer.

Androgen deprivation therapy (ADT) remains the cornerstone of metastatic prostate cancer treatment and is typically administered continuously and indefinitely. While effective in controlling disease, prolonged testosterone suppression has wide-ranging physiological consequences, affecting bone health, cardiovascular risk, and possibly mood, physical, and cognitive function. Moreover, significant improvements in survival due to combination and intensification strategies have led to prolonged treatment and cumulative exposure to treatment-related toxicities. Consequently, metastatic prostate cancer is increasingly a chronic condition for many patients and represents a growing area of survivorship challenge.

## 2. The Changing Landscape of Metastatic Prostate Cancer

The therapeutic landscape of metastatic prostate cancer has undergone substantial transformation over the past decade [3]. Historically, treatment was largely limited to ADT. While this produced some survival benefits, the introduction of combinations of systemic therapies incrementally improved outcomes for both hormone-sensitive and castration-resistant metastatic disease [3,4].

### 2.1. Metastatic Hormone-Sensitive Prostate Cancer

In metastatic hormone-sensitive prostate cancer (mHSPC), several landmark randomized trials have established the benefit of early treatment intensification (Table 1). The first major treatment advances leading to improved survival were the addition of docetaxel to ADT in the CHAARTED and STAMPEDE trials [5,6,7,8,9].

Despite suppression of gonadal testosterone production with ADT, prostate cancer may continue to be driven by adrenal androgens, intratumoral steroidogenesis, or androgen receptor mutations. To overcome these cancer escape mechanisms, androgen receptor pathway inhibitors (ARPIs) in combination with ADT were introduced, providing more complete pathway suppression [19]. Several phase III trials, including the LATITUDE trial for abiraterone, the ENZAMET trial and the ARCHES trial for enzalutamide, and the TITAN trial for apalutamide, demonstrated substantial overall survival benefits of more than 5 years with the addition of ARPIs to ADT [10,11,12,13,14,15,20,21,22,23]. However, despite a significant progression-free survival benefit, ARANOTE for darolutamide has not yet demonstrated a mature overall survival benefit, possibly due to the availability of multiple effective subsequent life-prolonging therapies in contemporary clinical practice [16].

ADT plus ARPI is the standard of care for most patients with mHSPC and has largely replaced ADT plus docetaxel due to its broad efficacy and favorable tolerability. To date, no specific ARPI has demonstrated clear oncological superiority over another, and treatment selection is therefore guided by toxicity profiles, comorbidities, potential drug–drug interactions, and patient preferences.

Further intensification with triplet therapy has also improved outcomes. The PEACE-1 and ARASENS trials demonstrated that adding abiraterone or darolutamide, respectively, to ADT plus docetaxel further improved overall survival, particularly in patients with high-volume or high-risk disease [17,18]. Consequently, triplet therapy has become an established option for fit patients with substantial disease burden.

Beyond systemic treatment intensification, local and metastasis-directed therapies (MDT) have emerged as additional strategies in selected patients with oligometastatic disease. Although their long-term oncological impact remains under investigation, studies such as STAMPEDE, STOMP, and ORIOLE suggest that these approaches may delay disease progression, delay the need for systemic therapies, or reduce local tumor-related complications [24,25,26,27]. From a survivorship perspective, reducing exposure to long-term systemic therapy may also postpone treatment-related toxicities. However, current research is increasingly focusing on multimodal approaches that combine MDT with a finite course of systemic therapy. The phase II RADIOSA trial demonstrated improved disease control with SBRT plus short-term ADT compared with SBRT alone, while the phase II PEACE V-STORM trial showed improved metastasis-free survival with elective pelvic nodal irradiation and a simultaneous boost to PSMA PET/CT-positive lymph nodes compared with MDT alone, without a relevant increase in toxicity [28,29]. The value of these strategies is being further investigated in ongoing prospective trials (Supplemental Table S1).

### 2.2. Metastatic Castration-Resistant Prostate Cancer

Despite modern combination therapies, many patients eventually develop castration-resistant disease. Over the past decade, the management of metastatic castration-resistant prostate cancer (mCRPC) has evolved into an increasingly sequential and individualized treatment paradigm, with treatment selection guided by prior therapies in the mHSPC setting, as well as performance status, comorbidities, metastatic distribution, and molecular characteristics. The widespread use of ARPIs in mHSPC has altered treatment sequencing in mCRPC, as cross-resistance often limits the benefit of sequential ARPI therapy (Table 2).

Taxane-based chemotherapy remains a key component of mCRPC management, with docetaxel representing an important option for chemotherapy-naïve patients and cabazitaxel demonstrating better survival to an alternative ARPI after docetaxel in the CARD trial [30]. Another major advance has been the introduction of poly (ADP-ribose) polymerase (PARP) inhibitors. Initially, they were investigated in patients with homologous recombination repair (HRR) gene alterations and more recently in combination with ARPIs, as demonstrated in trials such as PROpel, TALAPRO-2, and MAGNITUDE [31,34,35,36,37,39,40].

PSMA-targeted radioligand therapy has further expanded treatment options. The VISION and PSMAfore demonstrated clinical benefit with lutetium-177–PSMA-617 in previously chemo-treated and chemo-naïve patients, respectively, with PSMA-positive mCRPC [32,33]. Radium-223 also remains an option for selected patients with symptomatic bone-predominant mCRPC, with or without enzalutamide [38,41,42]. Together, these developments have transformed mCRPC from a disease with limited treatment options into a setting characterized by multiple effective sequential therapies.

While all these advances have significantly prolonged survival, they have also increased the duration and complexity of treatment exposure. Consequently, we are facing a growing population of patients living for many years with metastatic prostate cancer and the cumulative consequences of long-term systemic therapy.

## 3. Prostate Cancer Survivorship in the Context of Metastatic Disease

Survivorship in prostate cancer has traditionally been framed in the context of localized disease and curative treatment, with emphasis on long-term outcomes following definitive therapy [43,44]. In metastatic prostate cancer, however, survivorship represents a fundamentally different clinical scenario. It is characterized by persistent disease and variable disease burden. Patients are exposed to extended durations of systemic therapy and multiple sequential treatment lines. Therefore, there is a need to balance disease control with treatment toxicity and quality of life.

Recent Multinational Association of Supportive Care in Cancer (MASCC)–ASCO standards and practice recommendations (which are not specific to prostate cancer) reflect the paradigm that survivorship extends beyond crude survival alone [45]. They highlight the importance of longitudinal care, integration of patient-reported outcomes, and the multidisciplinary management of physical, psychological, and social needs [45]. Moreover, the National Comprehensive Cancer Network (NCCN) Survivorship guidelines acknowledge that there is a growing population with chronic and/or metastatic disease that is included in the targeted population [46]. The NCCN Prostate Cancer guidelines include principles of survivorship with an emphasis on bone and cardiovascular health for patients with prostate cancer, especially on ADT [47]. The European Association of Urology (EAU) Prostate Cancer guideline also emphasizes the importance of balancing treatment risks and benefits for men with prostate cancer [48]. Moreover, the Prostate Cancer Working Group 4 recommends incorporating additional patient-reported outcomes and reliable assessment of post-treatment outcomes into metastatic prostate cancer trials [49].

## 4. Treatment-Related Toxicities: A Multidimensional Burden

### 4.1. Bone Health and Skeletal Complications

The adverse effects of ADT on bone health are well established. ADT increases bone remodeling, leading to a reduction in bone mineral density and consequently an increased risk of fractures. In prostate cancer patients treated with ADT, the relative risk of fractures has been reported to increase by up to 45% [50]. These fractures are associated with increased morbidity and mortality [51]. In the current metastatic prostate cancer setting, however, bone health is no longer only about preventing skeletal-related events in end-stage disease. With significantly improved survival, it has become a longitudinal survivorship issue requiring ongoing attention to cumulative skeletal toxicity and optimization of bone-protective strategies over time. This includes considerations regarding the timing, combinations, and duration of bone-protective therapies.

Several landmark trials have established the role of bone-modifying agents in mCRPC. They demonstrated the efficacy of zoledronic acid and denosumab in reducing skeletal-related events [52]. Consequently, international guidelines consistently recommend the use of bone-protective agents in patients with mCRPC and bone metastases [47,48]. More recent investigations have shifted the focus towards bone health in the context of modern treatment intensification. The ERA-223 trial highlighted an increased fracture risk when radium-223 was combined with abiraterone in the absence of adequate bone protection [53]. The trial was stopped due to increased rates of fractures and death without improved symptomatic skeletal event-free and overall survival. In contrast, in the later PEACE-3 trial (enzalutamide with or without radium-223), bone-protecting agents with denosumab or zoledronic acid were required. Significantly reduced fracture rates were observed, underscoring the importance of integrating bone protection into contemporary treatment strategies [54]. The recent phase II COMRADE study evaluating olaparib in combination with radium-223 in patients with mCRPC and bone metastases demonstrated encouraging clinical activity [55]. As treatment strategies increasingly incorporate bone-targeted agents, proactive bone health management remains essential to minimize skeletal complications and preserve quality of life.

With increasing survival and prolonged systemic treatment duration, the optimal timing of bone-protecting agents is uncertain because their most important toxicities (osteonecrosis of the jaw, hypocalcemia, atypical femoral fractures, renal toxicity) are duration-dependent. The REDUSE trial is therefore of particular interest, investigating whether denosumab can be safely de-escalated from every 4 weeks, which is the current standard in the mCRPC setting, to every 12 weeks in patients with mCRPC in order to reduce treatment burden without compromising efficacy. At ASCO 2026, the REDUSE trial findings provided evidence that 12-weekly denosumab maintained efficacy in preventing symptomatic skeletal events while reducing treatment burden and cumulative toxicity, including osteonecrosis of the jaw and hypocalcemia [56].

Despite guideline recommendations of bone health assessment and management in mCRPC, observational data suggest that bone-protective agents remain underutilized in eligible patients, highlighting an important gap between evidence and clinical practice [57].

Also, the role of bone-protective therapy in earlier disease stages remains less clearly defined. Since there is no consistent evidence of their efficacy in the context of ADT monotherapy for mHSPC [58], there is no current role for denosumab or zoledronic acid in patients with mHSPC receiving ADT in isolation [47,48]. However, recent meta-analyses have shown higher fracture rates with intensified mHSPC treatment using ADT plus ARPIs, with or without docetaxel [59]. This shift underscores the need for renewed attention to skeletal health in patients receiving contemporary treatment regimens for mHSPC. Although evidence from STAMPEDE and LATITUDE substudies is limited to secondary and post hoc analyses, they suggested that bone-modifying agents may reduce fracture-related hospitalizations and delay skeletal-related events in patients receiving contemporary systemic therapy for mHSPC [60,61].

Prospective support comes from the randomized phase II BONENZA trial in patients treated with ADT plus enzalutamide, in which zoledronic acid improved mineral density, bone microarchitecture, and bone turnover markers [62]. However, no significant changes in bone response in whole-body diffusion-weighted magnetic resonance imaging (primary outcome) were demonstrated, and oncological benefit remains uncertain [63]. These findings suggest that bone-protective agents may also contribute to long-term skeletal preservation in patients exposed to prolonged treatment intensification in mHSPC. However, these developments could mean that patients will be on long-term bone protection treatment, not only in later stages such as mCRPC but also in earlier stages, with associated duration-dependent toxicities.

The most important trials are summarized in Supplemental Table S2.

### 4.2. Cardiovascular Effects

Cardiovascular disease (CVD) is highly prevalent among men with prostate cancer and represents a major contributor to both morbidity and mortality [64]. Multiple observational studies and meta-analyses have demonstrated an association between ADT and increased cardiovascular risk, including higher rates of hypertension, diabetes, coronary artery disease, myocardial infarction, stroke, and sudden cardiac death [65,66,67,68]. The risk appears greatest during the first months after treatment initiation and among patients with pre-existing cardiovascular disease [67]. Cardiovascular disease is also a leading cause of death in men with prostate cancer, ranking second only to prostate cancer-specific mortality in patients with metastatic disease [69]. While observational studies have suggested an association between ADT and cardiovascular mortality, randomized clinical trials have not consistently confirmed this relationship [4]. This likely reflects residual confounding in observational datasets and the underrepresentation of patients at high cardiovascular risk in clinical trials.

To address the lack of prospective cardiovascular data, the RADICAL PC program was established, consisting of a large observational registry (RADICAL PC-1 = The Role of Androgen Deprivation Therapy in CVD: a Longitudinal Prostate Cancer Study) and a nested randomized trial evaluating systematic cardiovascular and lifestyle risk-factor modification (RADICAL PC-2 = Role of Androgen Deprivation Therapy in CVD: Randomized Intervention for CV and Lifestyle Risk Factors in Prostate Cancer Patients) in patients with newly diagnosed prostate cancer or starting ADT for the first time [70]. Early findings from the RADICAL PC study showed that 69% of patients with prostate cancer were at high cardiovascular risk based on the Framingham Risk Score, with ADT independently associated with high baseline cardiovascular risk in multivariable analyses [71]. In addition, 99% of patients had at least one uncontrolled modifiable cardiovascular risk factor, and 51% exhibited suboptimal control of most of the modifiable cardiovascular risk factors [72].

Given the cardiovascular concerns associated with ADT, increasing attention has focused on potential differences in cardiovascular safety between gonadotropin-releasing hormone (GnRH) agonists and antagonists. The PRONOUNCE trial was unable to demonstrate any difference between degarelix (GnRH antagonist) and leuprolide (GnRH agonist) because of premature termination and limited statistical power [73]. The HERO trial reported lower rates of major adverse cardiovascular events (MACE) with the oral GnRH antagonist relugolix compared with leuprolide, as a prespecified safety endpoint [74,75]. Supporting these findings, the REVELUTION study demonstrated less progression of coronary plaque volume among patients treated with relugolix compared to leuprolide [76]. Therefore, current evidence suggests a potential cardiovascular advantage of GnRH antagonists, especially in prostate cancer patients with established cardiovascular disease [77]. However, adequately powered cardiovascular outcome trials remain necessary to confirm this observation. Importantly, most available cardiovascular data originate from studies including non-metastatic or mixed-stage populations. Moreover, these studies were largely conducted in the era of ADT monotherapy. For patients with metastatic prostate cancer, cardiovascular toxicity may be particularly important since systemic treatment is typically administered lifelong to a particularly high-risk population [78], although potential benefits might be attenuated because of the competing risk of prostate cancer death.

The most important trials are summarized in Supplemental Table S3.

### 4.3. Physical Function and Body Composition

Alongside cardiovascular risk, ADT is associated with alterations in body composition, including increased fat mass, reduced lean body mass, sarcopenia, and weight gain. In the RADICAL PC cohort, ADT was associated with a 1.6% increase in weight, a 2.2% increase in waist circumference, and a 27.4% reduction in handgrip strength over 12 months [79]. High waist circumference (>110 cm, HR 1.4, 95% CI 1.03–1.90) and low handgrip strength (<29.5 kg; HR 1.59, 95% CI 1.14–2.22) were independently associated with incident MACE. Strategies aimed at preserving physical function have become an increasingly important component of survivorship care in men with prostate cancer.

Non-pharmacological strategies are gaining increasing attention, especially structured exercise programs. Randomized trials have demonstrated that aerobic, resistance, and combined exercise programs improve physical function, muscle strength, cardiorespiratory fitness, body composition, and health-related quality of life in men undergoing ADT. Evidence investigating immediate versus delayed exercise demonstrated that initiating exercise concurrently with ADT attenuated treatment-related adverse effects. These findings suggest that exercise may serve not only as a rehabilitative intervention but also as a preventive strategy when initiated at the start of ADT [80,81,82,83]. More recent trials have further demonstrated that progressive resistance training can effectively counteract ADT-associated sarcopenia, resulting in significant gains in lean body mass and muscle strength [84,85]. Combined exercise and dietary interventions improved endothelial function in addition to musculoskeletal benefits [86,87]. It remains to be determined whether these benefits are associated with favorable effects on cardiovascular and metabolic health. Supervised exercise remained effective in patients receiving intensified hormonal therapy with enzalutamide, resulting in improved fitness and physical performance [88]. Emerging evidence also suggests that behavioral and lifestyle interventions may facilitate long-term physical activity participation, highlighting the importance of motivation, self-management, and accessible delivery models such as home-based exercise programs [89,90,91].

Despite the evidence supporting exercise during ADT, relatively few studies have focused specifically on metastatic prostate cancer. Among the available data, a pilot study in men with metastatic castration-resistant prostate cancer demonstrated that aerobic and resistance exercise was feasible and associated with improvements in several quality-of-life domains [92]. Furthermore, a randomized trial including both localized and metastatic patients receiving ADT reported no exercise-related adverse events and demonstrated improvements in strength and emotional functioning, suggesting the safety of supervised exercise in advanced disease settings [93]. Ongoing research continues to explore exercise interventions in men with mHSPC, including studies targeting patients with bone metastases and those at increased cardiovascular risk [94,95]. Finally, the recently initiated STAMINA trial is investigating the clinical effectiveness, implementation, and cost-effectiveness of exercise-based survivorship interventions in the routine care of prostate cancer patients on ADT [96].

Beyond exercise, pharmacological approaches targeting obesity and unfavorable changes in body composition are attracting increasing interest. Incretin mimetics, including glucagon-like peptide-1 receptor agonists (GLP-1RAs) and dual glucose-dependent insulinotropic polypeptide/glucagon-like peptide-1 (GIP/GLP-1) receptor agonists, have demonstrated substantial weight loss in large, randomized trials conducted in patients with obesity [97,98,99]. In addition, these agents improve glycemic control and several cardiometabolic risk factors, while semaglutide has also been shown to reduce major adverse cardiovascular events in individuals with overweight and established cardiovascular disease [99,100,101,102]. Given the high prevalence of ADT-associated weight gain and cardiovascular risk, these findings are very relevant to men with prostate cancer. However, in most of these trials, cancer patients were excluded, and evidence for incretin mimetics in prostate cancer populations remains limited [103]. Dedicated studies are now underway, including the GAIN-PC CONTROL (NCT06908694) study evaluating semaglutide in men receiving ADT and the randomized phase II IMPACT-ADT trial (NCT07202247) comparing GLP-1-based therapy with dietary interventions to improve cardiometabolic health during hormone therapy.

Although many ADT-related functional impairments appear independent of disease stage, patients with metastatic prostate cancer often face additional challenges. These include potentially higher symptom burden, treatment intensification, and greater comorbidity. Moreover, metastatic disease may be associated with cancer-related cachexia and progressive weight loss, raising concerns that interventions promoting weight reduction, such as incretin mimetics, could also have unintended consequences in some patients.

### 4.4. Vasomotor Symptoms

Vasomotor symptoms, including hot flushes and night sweats, are among the most common adverse effects of ADT. These symptoms negatively impact sleep quality, daily functioning, and health-related quality of life. They are particularly relevant in men with metastatic prostate cancer, for whom ADT is typically administered lifelong. Despite their clinical significance, vasomotor symptoms remain relatively understudied compared with other survivorship domains. Recent work emphasized the relative invisibility of this treatment-related toxicity, which has been the subject of fewer therapeutic advances compared with those achieved in women’s menopausal vasomotor symptoms [104]. Several pharmacological and non-pharmacological interventions have been evaluated for the management of ADT-related vasomotor symptoms.

Among non-pharmacological approaches, cognitive behavioral therapy has emerged as a promising strategy, with the MANCAN2 randomized trial demonstrating significant short-term reductions in the burden and distress associated with hot flushes and night sweats in men receiving ADT [105]. Complementary approaches such as acupuncture are also being investigated, with the ongoing AVAIL trial evaluating acupuncture compared with sham acupuncture and usual care [106].

Novel pharmacological interventions are likewise being explored. A recent randomized trial suggested that melatonin may reduce hot-flush severity [107]. In addition, agents such as gabapentin, venlafaxine, oxybutynin, and fezolinetant have been and continue to be under investigation. Gabapentin has been investigated in a randomized, double-blind, placebo-controlled phase III trial in prostate cancer on ADT, showing a significant reduction in hot flash burden compared with placebo, even in longer-term follow-up [108]. Moreover, NCT01533753 is a randomized clinical trial that compares gabapentin and venlafaxine for treating hot flashes in patients with prostate cancer on ADT [109]. Recent evidence from the phase III Alliance A222001 trial demonstrated that oxybutynin significantly reduced hot flash frequency and severity compared with placebo in men receiving ADT for prostate cancer [110]. Finally, fezolinetant, a selective neurokinin 3 receptor antagonist approved for the treatment of moderate-to-severe vasomotor symptoms in menopausal women, is currently under investigation in men with prostate cancer on ADT [111].

Beyond symptomatic management, emerging evidence suggests that the choice of androgen-suppression strategy itself may influence the development of vasomotor symptoms. The PATCH trial demonstrated substantially lower rates of hot flushes with transdermal estradiol compared with GnRH agonists, supporting the hypothesis that maintaining physiologic estrogen levels despite testosterone suppression may reduce ADT-related vasomotor symptoms. However, transdermal estradiol is associated with increased rates of gynecomastia and requires careful patient selection [112]. Despite representing one of the most common adverse effects of ADT, hot flushes remain a comparatively overlooked aspect of prostate cancer survivorship care. Addressing this gap will require greater recognition of vasomotor symptoms as a clinically meaningful toxicity and further research into supportive care for men receiving long-term ADT.

### 4.5. Patient-Reported Quality of Life and Psychological Health

Patient-reported outcomes (PROs) have become increasingly incorporated into metastatic prostate cancer trials, offering important insights into the patient experience. Across landmark studies evaluating treatment intensification, improvements in survival were generally achieved without compromising overall health-related quality of life (HRQoL).

Specifically, in the mHSPC setting, most randomized trials demonstrated maintenance of overall HRQoL or delayed deterioration rather than clear improvements compared with ADT alone. While LATITUDE reported improvements across multiple quality-of-life measures, studies such as TITAN and ARCHES demonstrated limited differences in HRQoL outcomes despite significant oncological benefit [15,113,114,115]. More recently, darolutamide-based regimens evaluated in ARASENS and ARANOTE were associated with clinically meaningful delays in pain progression and HRQoL deterioration [16,18,116].

In the mCRPC setting, several therapies have demonstrated favorable patient-reported outcomes. Radium-223 (ALSYMPCA), olaparib (PROfound), and [177Lu] Lu-PSMA-617 (VISION and PSMAfore) were associated with delayed deterioration in HRQoL and improvements in pain-related outcomes [117,118,119]. However, interpretation of these results requires caution when considering psychological survivorship. Most pivotal trials assessed psychological well-being indirectly through multidimensional PRO instruments such as Functional Assessment of Cancer Therapy–Prostate (FACT-P), Brief Pain Inventory–Short Form (BPI-SF), European Organization for Research and Treatment of Cancer Quality of Life Questionnaire Core 30 (EORTC QLQ-C30), and EuroQol 5-Dimension (EQ-5D) rather than through dedicated assessments of mental health. This limitation is illustrated by ENZAMET, in which enzalutamide was associated with increased fatigue and cognitive symptoms, yet overall deterioration-free survival for HRQoL favored treatment intensification because of superior disease control [120].

The Setting International Standards in Analyzing Patient-Reported Outcomes and Quality of Life Endpoints in Cancer Clinical Trials-Innovative Medicines Initiative (SISAQOL-IMI) characterizes the magnitude of changes in PRO score over time [121]. Furthermore, a key methodological consideration in metastatic prostate cancer trials is the heterogeneity in the timing and frequency of PRO assessments. Schedules vary widely between the trials, and these choices can fundamentally change the conclusions as laid out by Di Maio in a recent review [122]. For example, in CHAARTED, ADT plus docetaxel showed a transient QoL decline at 3 months, followed by recovery or even superiority at 12 months [123]. Less frequent early assessment may have missed this pattern.

The SISAQOL-IMI emphasizes the importance of missing PRO data since they are almost never random [121]. Sicker, progressing, or dying patients are more often lost to follow-up. This means complete-case analyses are biased with the risk of overestimating treatment benefit. In a systematic review about PRO data reporting and analysis in genitourinary cancers, 52% of the trials reported questionnaire completion and compliance rates at baseline and follow-up [124]. And although in 70% of the trials the protocol described the planned approach to handling missing data, only 48% specified multiple approaches. Haslam et al. found that only 5 oncology trials (3.4%) assessed QoL until death, including only one in metastatic or incurable cancers [125]. Among the five that did, only one reported a positive QoL outcome from the intervention. This observation suggests that truncating PRO collection at treatment discontinuation or radiographic progression may systematically overestimate treatment benefit by excluding the period of greatest QoL decline.

As patients live longer with mHSPC, QoL data increasingly guide decisions on whether a regimen is sustainable over the years. If PRO collection stops at progression, the data misses the phase with the highest symptom burden and therefore the phase most relevant for survivorship and end-of-life care. If QoL is only reported as mean change at fixed time points, transient declines may look the same as sustained decline. This can lead to under- or overestimation of the true burden. Informative censoring adds to this problem. For survivorship care, this means current PRO evidence may not reliably capture late-emerging toxicities in patients living for years with metastatic disease.

Dedicated assessments of anxiety, depression, fear of progression, uncertainty regarding prognosis, cognitive concerns, and caregiver burden are largely absent from trials. All of this applies despite the fact that the NCCN survivorship guidelines specifically recommend tools such as the Patient Health Questionnaire-9 (PHQ-9), Generalized Anxiety Disorder-7 (GAD-7), and Primary Care Posttraumatic Stress Disorder Screen for Diagnostic and Statistical Manual of Mental Disorders (PC-PTSD-5) for depression, anxiety, distress, and trauma screening in cancer survivors [46]. Moreover, they present comprehensive flowcharts for screening of depression, anxiety, panic, trauma, and distress while integrating non-pharmacologic and pharmacologic interventions. These screening tools remain unused as endpoints in randomized controlled trials for metastatic prostate cancer, although available evidence suggests that depression represents a non-negligible burden among patients with prostate cancer [126]. A 2021 systematic review and meta-analysis reported that approximately 6% met the criteria for depressive disorders and 17% experienced significant depressive symptoms in self-reporting tools [127]. Moreover, approximately 17% had significant anxiety symptoms, and nearly 10% reported recent suicidal ideation. Population-based data from the SEER-Medicare database reported that ADT was independently associated with increased risk for depression (HR 1.23; 95% CI 1.15–1.31) and inpatient psychiatric treatment (HR 1.29; 95% CI 1.17–1.41) compared to prostate cancer patients not receiving ADT. Notably, the risk for depression appeared duration-dependent, increasing by 12% among patients receiving ADT for ≤6 months and by 37% among patients treated for ≥12 months [128]. Consistent with these findings, a more recent meta-analysis reported 46% increased odds of depression among prostate cancer patients using ADT [129]. Despite this burden, data from the Clinformatics Data Mart Database (Optum) indicated that nearly 50% of prostate cancer patients diagnosed with depression while receiving ADT had not documented mental health treatment [130]. Ongoing studies are specifically addressing the mental health needs of men with metastatic prostate cancer. Prospective international registries such as the IRONMAN (NCT03151629), which is enrolling 5000 men with mHSPC and CRPC across 16 countries, may help further characterize psychological health impairments of metastatic prostate cancer [131].

Multidisciplinary support models play a critical role in addressing the psychosocial burden associated with metastatic prostate cancer. A systematic review of 22 RCTs found that psychological interventions significantly reduced depression, anxiety, and distress in prostate cancer, and especially that combined cognitive- and education-based approaches may derive greater benefit. However, most trials enrolled men with localized prostate cancer, and evidence specific to metastatic or advanced disease was limited [132]. In advanced prostate cancer specifically, the evidence is mixed. An RCT (N = 189) of telephone-delivered mindfulness-based cognitive therapy in patients with advanced prostate cancer found no significant benefit over minimally enhanced usual care, suggesting that generic mindfulness approaches may not adequately address the needs of this population [133]. A technology-assisted psychosocial RCT in advanced prostate cancer (N = 192), evaluating cognitive-behavioral stress management (CBSM), did not significantly improve overall health-related quality of life or symptom burden compared with an active health promotion control [134]. However, a secondary analysis of the same trial found that among men with low baseline social well-being, CBSM significantly reduced fear of cancer recurrence and cancer-related intrusive thoughts, with benefits sustained at 12 months, suggesting that tailored interventions targeting specific psychosocial vulnerabilities may be more effective than broad-spectrum approaches [135]. An ongoing interventional trial is testing targeted psychosocial approaches in this population. The ENGAGE trial (NCT06555588) is evaluating a telehealth-delivered cognitive-behavioral/acceptance-based intervention to reduce pain-, fatigue-, and distress-related symptom interference in patients with stage IV prostate and other solid tumors. Beyond psycho-oncology-specific interventions, early systematic integration of palliative care has demonstrated significant improvements in quality of life in advanced cancer, though prostate cancer-specific trials are lacking [136]. Peer support interventions have shown positive effects on quality of life, depression, and anxiety across cancer types, but no RCT has been conducted in the metastatic prostate cancer population [137].

As survival continues to improve and patients increasingly live for many years with metastatic disease, understanding the psychological consequences of prolonged treatment exposure and chronic disease management may become even more important. Further research is therefore essential to address this unmet need, especially in metastatic prostate cancer.

## 5. Future Directions in Survivorship-Oriented Care

As survival continues to improve, focus on optimizing long-term survivorship should accompany research on treatment intensification. One emerging area of interest is the development of risk-adapted treatment strategies, including treatment interruption, treatment de-escalation, and individualized treatment duration. The SWOG S9346 trial failed to establish the non-inferiority of intermittent ADT compared with continuous ADT in mHSPC [138]. However, the contemporary treatment landscape has fundamentally changed. The current standard with ADT and ARPIs (with or without docetaxel) results in deeper and more durable responses. Subgroup analyses from landmark trials such as TITAN and ARCHES have demonstrated particularly favorable outcomes among patients achieving profound prostate-specific antigen responses. Therefore, an unresolved question is whether selected exceptional responders can safely reduce or interrupt treatment [13,139,140]. This concept is currently being investigated in several prospective studies. The phase II A-DREAM study enrolled patients with mHSPC who achieved a deep response following prolonged treatment with ADT plus ARPI. Preliminary results were presented at ASCO 2026 (full publication pending) [141]. After treatment discontinuation, 41% of patients remained treatment-free with recovery of eugonadal testosterone levels at 18 months (primary endpoint). At a median follow-up of 26.9 months, 38.5% remained off treatment, and the median treatment-free interval was 24.5 months. Importantly, most patients who restarted therapy were successfully re-treated with their original ADT plus ARPI regimen. Only one of four deaths observed during follow-up was attributable to prostate cancer. This again emphasizes the growing importance of competing health risks and treatment-related morbidity in prostate cancer patients experiencing prolonged survival. The ongoing phase III EORTC 2238 De-Escalate trial is evaluating intermittent versus continuous maximum androgen blockade (ADT+ARPI) in patients with mHSPC achieving a prostate-specific antigen level ≤ 0.2 ng/mL after initial therapy and will provide important data regarding oncological outcomes, toxicity, and health-related quality of life [142]. Similarly, the phase II OPTIMAS trial (NCT07216248) is investigating intermittent treatment strategies using relugolix-based regimens. The LIBERTAS study (NCT05884398) is exploring ADT-sparing approaches in mHSPC.

These de-escalation trials share a common rationale for evaluating whether patients with deep PSA response (≤0.2 ng/mL) on ADT+ARPI may safely de-intensify treatment. However, the trial designs differ in several key aspects. The required duration of initial treatment varies. A-DREAM requires 540–750 days of ADT plus ≥ 360 days of ARPI, with three consecutive PSA values below 0.2 ng/mL and testosterone < 50 ng/dL. This selects only the most exceptional responders. EORTC 2238 De-Escalate allows randomization if PSA ≤ 0.2 ng/mL 6 to 12 months after start of treatment, and for LIBERTAS, it is 6 months. OPTIMAS does not specify a fixed duration at all. The trials also differ in what is actually interrupted. A-DREAM and EORTC 2238 De-Escalate stop both ADT and ARPI completely—a full treatment holiday. OPTIMAS interrupts both relugolix and ARPI intermittently. LIBERTAS is different since it continues apalutamide and interrupts only ADT. Testosterone recovery, therefore, happens under ongoing AR blockade. This is a fundamentally different biological strategy.

Monitoring protocols vary as well. A-DREAM is highly structured, with PSA and testosterone every 3 months, imaging every 6 months, and FACT-P every 6 months. EORTC 2238 De-Escalate is more pragmatic and less prescriptive. LIBERTAS adds digital health tools measuring sleep, activity, and neurocognitive function, and patient-reported outcomes, including physical and mental well-being, alongside standard PSA and imaging follow-up. Re-initiation thresholds are inconsistent, too. A-DREAM restarts treatment at PSA ≥ 5 ng/mL, radiographic changes, or PC-related symptoms. LIBERTAS restarts in case of new/worsening cancer symptoms, at PSA > 10 ng/mL or PSA doubling time <6 months. For EORTC 2238 De-Escalate, the decision to restart treatment is left to the investigator’s discretion. Finally, the primary endpoints reflect different priorities. EORTC 2238 De-Escalate is the only trial powered for overall survival non-inferiority, making it the key efficacy trial. LIBERTAS uses co-primary endpoints of 18 months radiographic progression-free survival and hot flash burden. OPTIMAS focuses on patient-reported fatigue (Brief Fatigue Inventory, BFI-3) in Cohort A. A-DREAM uses treatment-free survival with testosterone recovery (>150 ng/dL) at 18 months.

Combination therapy remains the current standard of care. Most clinicians would still favor guideline-recommended treatment intensification at this point. However, the de-escalation trials point to a broader shift. Instead of intensification as the default, future strategies may weigh oncological efficacy against long-term quality of life. This matters for survivorship. As more patients live for years with mHSPC, the cumulative burden of continuous ADT and ARPI becomes an important factor for long-term well-being. De-escalation is essentially an attempt to regain treatment-free time without compromising survival. However, the trial heterogeneity reflects an unresolved question of whether complete treatment holidays in exceptional responders or selective ADT interruption with continued ARPI in a broader population will balance oncological efficacy against long-term quality of life. These are two different approaches to the same goal. This may need to be resolved before de-escalation can enter clinical guidelines in the future. The primary endpoints reflect this divergence, too. Each captures a different part of survivorship. EORTC 2238 De-Escalate is powered for overall survival non-inferiority. This gives the clearest answer on oncological safety but says little about the quality of the years gained. A-DREAM uses treatment-free survival with testosterone recovery. This reflects survivorship more directly, since eugonadal status is linked to bone health and metabolic profile. However, it does not confirm long-term safety. LIBERTAS combines radiographic progression-free survival with hot flash burden, pairing efficacy with one specific and important toxicity. OPTIMAS prioritizes patient-reported fatigue, treating a single symptom as being as clinically meaningful as tumor control while leaving oncological differences as a secondary consideration. In summary, no single trial captures the full survivorship picture, and future guidance will need to combine evidence across these endpoints.

Moreover, advances in molecular characterization could support this shift toward more personalized survivorship care. Beyond informing treatment choice, biomarkers may eventually flag patients at higher risk of treatment-related toxicity, allowing supportive care and surveillance to be tailored rather than standardized. For example, monitoring for cardiometabolic, bone, or cognitive effects could be adjusted in intensity and frequency to a patient’s molecular and clinical risk profile, rather than following a fixed schedule for all patients. The goal would be a better balance between maximizing survival and limiting the cumulative burden of long-term systemic therapy.

## 6. Conclusions

**Figure 1:**
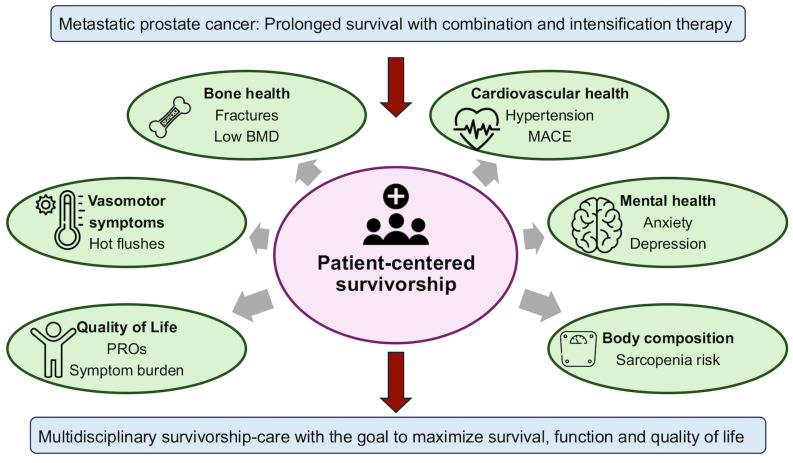
The expanding survivorship burden in metastatic prostate cancer. BMD = Bone mineral density; MACE = Major adverse cardiovascular events; PROs = Patient-reported outcomes.

Over the past decade, the therapeutic landscape of metastatic prostate cancer has changed substantially. Combination and intensification strategies have extended survival in both hormone-sensitive and castration-resistant disease. As a consequence, survivorship in this setting now has a different meaning than before. A growing number of men live for years, in some cases more than a decade, under continuous or near-continuous systemic therapy. Therefore, the clinical focus cannot remain on survival alone. It must also include the cumulative, long-term burden of treatment, including bone health, cardiovascular risk, body composition, vasomotor symptoms, and psychological well-being (Figure 1).

Across these domains, a similar pattern is seen. Evidence from localized or non-metastatic disease is often extrapolated to the metastatic setting. Dedicated trials in survivorship of metastatic disease remain limited, and patient-reported and psychological outcomes are not consistently assessed or reported. New strategies such as risk-adapted treatment de-escalation, biomarker-guided personalization, and structured exercise or cardiometabolic interventions may reduce this burden without reducing oncological efficacy. However, their implementation in clinical practice will require endpoints specific to survivorship, longer follow-up, and more representative trial populations than have been used so far.

In summary, survivorship in metastatic prostate cancer may warrant consideration as an important goal in its own right, rather than solely as a secondary outcome of treatment. Achieving this will likely require sustained research effort and multidisciplinary investment.

## Figures and Tables

**Table 1:** Randomized control trials in metastatic hormone-sensitive prostate cancer.

Trial Name	Intervention (n)	Comparator (n)	Median OS (Months)	HR (95% CI)	Median Follow-Up (Months)	Latest Update
CHAARTED [7,8]	Docetaxel + ADT (n = 397)	ADT (n = 393)	57.6 vs. 47.2	0.72 (0.59–0.89)	53.7	2025: 10-year OS 25.9% vs. 22.5%, HR 0.78 (0.66–0.93)
STAMPEDE [9] (Docetaxel, M1)	Docetaxel + ADT (n = 362)	ADT (n = 724)	NR vs. NR (M1 subgroup)	0.81 (0.69–0.95)	78.2	
LATITUDE [10]	Abiraterone + ADT (n = 597)	Placebo + ADT (n = 602)	53.3 vs. 36.5	0.66 (0.56–0.78)	51.8	
STAMPEDE [11] (Abiraterone)	ADT + abiraterone (n = 501)	ADT (n = 502)	76.6 vs. 45.7	0.62 (0.53–0.73)	96	
ENZAMET [12]	Enzalutamide + ADT (n = 563)	ADT + Nonsteroidal antiandrogen (n = 562)	NR vs. NR	0.70 (0.58–0.84)	68	2025: median OS 95 vs. 70 months, HR 0.73 (0.63–0.86) *
ARCHES [13,14]	Enzalutamide + ADT (n = 574)	Placebo + ADT (n = 576)	NR vs. NR	0.66 (0.53–0.81)	44.6	2026: 5-year OS 66% vs. 53%, HR 0.70 (0.58–0.85)
TITAN [15]	Apalutamide + ADT (n = 525)	Placebo + ADT (n = 527)	NR vs. 52.2	0.65 (0.53–0.79)	44.0	
ARANOTE [16]	Darolutamide + ADT (n = 446)	Placebo + ADT (n = 223)	NR vs. NR	0.81 (0.59–1.12)	25.3 vs. 25.0	
PEACE-1 [17] (ADT with docetaxel population)	Abiraterone + ADT + Docetaxel (n = 355)	ADT + Docetaxel (n = 355)	NR vs. 52.8	0.75 (0.59–0.95)	52.8	
ARASENS [18]	Darolutamide + Docetaxel + ADT (n = 651)	Placebo + Docetaxel + ADT (n = 654)	NR vs. 48.9	0.68 (0.57–0.80)	43.7 vs. 42.4	

* Abstract presented at ASCO 2025, full publication pending. Abbreviations: n = sample size, ADT = androgen deprivation therapy, OS = overall survival, HR = hazard ratio, CI = confidence interval, NR = not reached.

**Table 2:** Randomized control trials in metastatic castration-resistant prostate cancer.

Trial Name	Population	Intervention (n)	Comparator (n)	Median OS (Months)	HR (95% CI)	Median Follow-Up (Months)
ALSYMPCA [24]	Symptomatic bone lesions, no visceral metastases	Radium-223 (n = 614)	Placebo (n = 307)	14.9 vs. 11.3	0.70 (0.58–0.83)	- *
CARD [30]	Post-docetaxel and progressed ≤12 months on ARPI	Cabazitaxel + ADT (n = 129)	Second ARPI (n = 126)	13.6 vs. 11.0	0.64 (0.46–0.89)	9.2
PROfound [31] (Cohort A)	HRR-mutated (BRCA 1/2 or ATM) after ARPI	Olaparib (n = 162)	Second ARPI (n = 83)	19.1 vs. 14.7	0.69 (0.50–0.97)	21.9 vs. 21.0
VISION [32]	PSMA-avid lesions post-ARPI and post-docetaxel	Lu-177-PSMA-617 + SoC (n = 551)	SoC (n = 280)	15.3 vs. 11.3	0.62 (0.52–0.74)	20.9
PSMAfore [33]	PSMA-avid lesions post-ARPI, chemotherapy-naïve in mCRPC	Lu-177-PSMA-617 (n = 234)	Second ARPI (n = 234)	24.5 vs. 23.1	0.91 (0.72–1.14)	34.27
TRITON-3 [34] (BCRA)	BRCA-mutated, post-ARPI	Rucaparib (n = 201)	Physician’s choice (docetaxel or second ARPI) (n = 101)	24.3 vs. 20.8	0.81 (0.58–1.12)	-
PROPEL [35]	First line mCRPC, biomarker-unselected	Olaparib + abiraterone (n = 399)	Placebo + abiraterone (n = 397)	42.1 vs. 34.7	0.81 (0.67–1.00)	36.6 vs. 36.5
TALAPRO-2 [36]	First line mCRPC, biomarker-unselected	Talazoparib + enzalutamide (n = 402)	Placebo + enzalutamide (n = 403)	45.8 vs. 37.0	0.80 (0.66–0.96)	52.5
MAGNITUDE [37]	First line mCRPC (except up to 4 months of prior abiraterone allowed), HRR-alterations	Niraparib + abiraterone (n = 212)	Placebo + abiraterone (n = 211)	30.36 vs. 28.55	0.93 (0.72–1.20)	37.3
PEACE-3 [38]	First line mCRPC, chemotherapy-naïve in mCRPC, bone metastases	Enzalutamide + Radium-223 (n = 222)	Enzalutamide (n = 224)	38.2 vs. 32.6	0.76 (0.60–0.96)	58

* Terminated for efficacy at the prespecified interim analysis. Final overall survival presented at ASO GU 2025, full publication pending: for BRCA subgroup median follow-up 44 months, median OS 23.2 vs. 21.2 months, HR 0.91 (0.68–1.20). Abbreviations: n = sample size, mCRPC = metastatic castration-resistant prostate cancer, ADT = androgen deprivation therapy, ARPI = androgen receptor pathway inhibitor, HRR = homologous recombination repair, OS = overall survival, HR = hazard ratio, CI = confidence interval.

## Data Availability

No new data were created or analyzed in this study. Data sharing is not applicable to this article.

## Supplementary Materials

The following supporting information can be downloaded at: https://www.mdpi.com/article/10.3390/cancers18152465/s1, Table S1: Ongoing trial for oligometastatic hormone-sensitive prostate cancer; Table S2: Randomized control trials examining bone health and skeletal complications in prostate cancer; Table S3: Randomized control trials examining cardiovascular events in prostate cancer.

## Abbreviations

The following abbreviations are used in this manuscript:

ADT	Androgen deprivation therapy
mHSPC	Metastatic hormone-sensitive prostate cancer
ARPI	Androgen receptor pathway inhibitor
MDT	Metastasis-directed therapy
SBRT	Stereotactic body radiation therapy
PSMA PET/CT	Prostate-specific membrane antigen positron emission tomography/computed tomography
mCRPC	Metastatic castration resistant prostate cancer
PARP	Poly (ADP-ribose) polymerase
HRR	Homologous recombination repair
MASCC	Multinational Association of Supportive Care in Cancer
ASCO	American Society of Clinical Oncology
NCCN	National Comprehensive Cancer Network
EAU	European Association of Urology
CVD	Cardiovascular disease
GnRH	Gonadotropin-releasing hormone
HR	Hazard ratio
CI	Confidence interval
GLP-1RA	Glucagon-like peptide-1 receptor agonists
GIP/GLP-1	Glucose-dependent insulinotropic polypeptide/glucagon-like peptide-1
PROs	Patient-reported outcomes
HRQoL	Health-related quality of life
FACT-p	Functional Assessment of Cancer Therapy—Prostate
BPI-SF	Brief Pain Inventory (Short Form)
EORTC QLQ-C30	European Organization for Research and Treatment of Cancer Quality of Life Questionnaire Core 30
EQ-5D	EuroQol 5-Dimension
SEER	Surveillance, Epidemiology, and End Results
