# Supplementary material for: Survivorship Challenges in Metastatic Prostate Cancer in the Era of Prolonged Survival: A Review
Source: Cancers (Basel). 2026 Jul 31;18(15):2465. doi: 10.3390/cancers18152465 (PMC13465217; doi:10.3390/cancers18152465)
Supplement: Supplementary file 1 [file cancers-18-02465-s001.zip › cancers-4412558-supplementary.pdf]

Supplementary Materials

Content

Page 2-3: Supplemental Table S1: Ongoing trial for oligometastatic hormone-sensitive prostate cancer

Page 4-5: Supplemental Table S2: Randomized control trials examining bone health and skeletal complications in prostate cancer

Page: 6: Supplemental Table S3: Randomized control trials examining cardiovascular events in prostate cancer

Supplemental Table S1: Ongoing trial for oligometastatic hormone-sensitive prostate cancer				
**NCT number**	**Trial**	**Setting**	**Design**	**Primary Outcome**
NCT04787744	VA STARPORT	Oligometastatic mHSPC	Prospective, randomized, open-label, multi-center – standard systemic therapy with vs. without PET-directed local therapy	CRPC-free survival
NCT04983095	METRO	Oligometastatic mHSPC	Prospective, randomized, open-label, multi-center – standard care vs. SBRT+standard care	Failure free survival
NCT05939414	PSMA-DC	metachronous recurrent oligometastatic mHSPC	Prospective, randomized, open-label, multi-center – Lutetium (177Lu) Vipivotide Tetraxetan (AAA617) Versus Observation (SBRT to all lesions in both arms)	Metastasis Free Survival by conventional imaging
NCT05223803	TERPS	De Novo Oligometastatic mHSPC	Prospective, multi-center, - Best systemic therapy + primary prostate radiation + Stereotactic ablative radiation therapy as metastasis-directed therapy vs. Best systemic therapy + primary prostate radiation	2-year failure-free survival

NCT06378866	DIVINE	metachronous recurrent oligometastatic mHSPC	Prospective, randomized, open-label, multi-center - SBRT and 6 months ADT/ARPI followed by watchful wait vs. SBRT followed by watchful waiting (ADT/ARPI at progression)	radiographic progression-free survival
NCT04302454	ADOPT	metachronous recurrent oligometastatic mHSPC	Prospective, randomized, open-label, multi-center - metastasis-directed therapy alone vs. metastasis-directed therapy + 6-month ADT	Metastases progression-free survival
NCT03784755	PLATON	oligometastatic mHSPC	Prospective, randomized, open-label, multi-center – Standard of care + radiation to prostate vs. Standard of care + ablative radiation to all sites (prostate + all metastases)	Failure-free Survival
Abbreviations: NCT number = National Clinical Trial number; mHSPC = metastatic hormonsensitive prostate cancer; PET = Positron Emission Tomography; CRPC = castration-resistant prostate cancer; SBRT = Stereotactic Body Radiation Therapy; ADT = androgen deprivation therapy; ARPI = androgen-receptor pathway inhibitor.				

Supplemental Table S2: Randomized control trials examining bone health and skeletal complications in prostate cancer						
**Trial Name/ NCT number**	**Population**	**Intervention (n)**	**Comparator (n)**	**Median follow up**	**Outcome of Interest**	**Relevant Conclusions**
NCT00321620	mCRPC with bone metastasis that are bisphosphonate-native	Denosumab 120 mg SC q4 weeks (n=950)	Zoledronic acid 4 mg IV q4 weeks (n=951)	~12 months treatment	Time to first skeletal related event<br><br>20.7 months (18.8-24.9) for denosumab versus 17.1 months (15.0-19.4) for zoledronic acid<br><br>HR 0.82 (0.71-0.95)	Denosumab was superior to zoledronic acid for skeletal related events in mCRPC
ERA-223	mCRPC with asymptomatic or minimally symptomatic bone metastasis that are receiving abiraterone	Radium-223 + abiraterone (n=401)	Placebo + abiraterone (n=405)	~21 months	Symptomatic skeletal event-free survival<br><br>22.3 months (20.4-24.8) in radium-223 versus 26.0 (21.8-28.3) in placebo<br><br>HR 1.12 (0.92-1.37)	Trial terminated early due to increased fractures (29% versus 11%) and deaths in the intervention group. Do not use in this population.
PEACE-3	mCRPC with bone metastases and eligible for radium-223	Enzalutamide + radium-223 ± bone protective agents (i.e., denosumab or zoledronic acid) (n=222)	Enzalutamide (n=224) ± bone protective agents (i.e., denosumab or zoledronic acid)	Interim analysis	Secondary endpoint for symptomatic skeletal events 24% in combination arm versus 13% in enzalutamide arm.<br><br>NB, 55% and 57% of patients on bone protective agents in the combination and enzalutamide arms, respectively	Bone protective agents reduce fracture incidence, supporting routine bone-protective therapy with enzalutamide and radium-223

COMRADE	mCRPC and ≥2 bony metastases	Olaparib + radium-223 (n=61)	Radium-223 (n=59)	Interim analysis	Secondary endpoint for symptomatic skeletal events<br><br>12.7% in the combination arm versus 22.9% in the radium-223 arm<br><br>NB, ~90% of patients on bone protective agents in both groups	Reinforces that bone protective agents should be used with radium-223, including in the setting of a PARP-inhibitor

Abbreviations: NCT number = National Clinical Trial number; mCRPC = metastatic castration-resistant prostate cancer; SC = subcutaneous; IV = intravenous; q4 weeks = every 4 weeks; HR = Hazard ratio; PARP = Poly(ADP-ribose) polymerase

.

Supplemental Table S3: Randomized control trials examining cardiovascular events in prostate cancer

Trial Name	Population	Intervention (n)	Comparator (n)	Follow up	Outcome of interest	Relevant Conclusions
HERO	Advanced hormone-sensitive prostate cancer requiring ≥1 year of ADT (localized, locally advanced, recurrent or metastatic)	Relugolix (n=622)	Leuprolide (n=308)	Median 52 weeks	Major cardiovascular events<br><br>6.2% in the relugolix arm versus 2.9% in the leuprolide arm<br><br>HR 0.46 (0.24-0.88)	GnRH antagonists may be superior for patients with cardiovascular disease
PRONOUNCE	Prostate cancer with plans for at least 12 months of ADT and known atherosclerotic cardiovascular disease	Degarelix (n=275)	Leuprolide (n=269)	12 months analysis	Major cardiovascular events<br><br>5.5% in the degarelix arm versus 4.1% in the leuprolide arm<br><br>HR 1.28 (0.59-2.79)	Early termination of study due to underpowering and similar rates of MACE
REVELUTION	Localized prostate cancer treated with radiation and ADT	Relugolix (n=31)	Leuprolide (n=34)	12 months analysis	Change in coronary total plaque volume (CT)<br><br>Relugolix +25.0 mm^3 (-6-46 mm^3)<br>Leuprolide +56 mm^3 (19.5-156.6 mm^3)	GnRH agonists associated with increased in coronary artery total plaque volume compared to GnRH antagonists

Abbreviations: ADT = Androgen deprivation therapy; GnRH = Gonadotropin-releasing hormone; HR = Hazard ratio; MACE = Major adverse cardiovascular events; CT = Computed tomography.
